# Preventing Evolutionary Rescue in Cancer

**DOI:** 10.1101/2023.11.22.568336

**Published:** 2023-11-22

**Authors:** Srishti Patil, Yannick Viossat, Robert Noble

**Affiliations:** 1 Indian Institute of Science Education and Research, Pune, India; 2 Department of Mathematics, City, University of London, London, UK; 3 Ceremade, CNRS, Université Paris-Dauphine, Université PSL, Paris, France

**Keywords:** mathematical oncology, evolutionary therapy, evolutionary rescue, therapeutic resistance, cancer treatment

## Abstract

Extinction therapy aims to eradicate tumours by optimally scheduling multiple treatment strikes to exploit the vulnerability of small cell populations to stochastic extinction. This concept was recently shown to be theoretically sound but has not been subjected to thorough mathematical analysis. Here we obtain quantitative estimates of tumour extinction probabilities using a deterministic analytical model and a stochastic simulation model of two-strike extinction therapy, based on evolutionary rescue theory. We find that the optimal time for the second strike is when the tumour is close to its minimum size before relapse. Given that this exact time point may be difficult to determine in practice, we show that striking slightly after the relapse has begun is typically better than switching too early. We further reveal and explain how demographic and environmental parameters influence the treatment outcome. Surprisingly, a low dose in the first strike paired with a high dose in the second is shown to be optimal. As one of the first investigations of extinction therapy, our work establishes a foundation for further theoretical and experimental studies of this promising evolutionarily-informed cancer treatment strategy.

## 1 Introduction

Just as species in an ecosystem interact, compete for resources, adapt to changing environmental conditions and undergo natural selection, so cancer clones rise and fall in a tumour ecosystem. Darwinian principles inevitably determine therapeutic responses [1] including the emergence of resistance, which, despite pharmaceutical advances, remains the greatest challenge in oncology. As cancer cells can use a variety of adaptive strategies to achieve resistance [2], targeting a single molecular mechanism often proves ineffective in the long term [3]. Understanding intratumour evolutionary processes provides a rational foundation for developing treatment strategies that, by explicitly accounting for evolutionary dynamics, achieve better clinical outcomes [4, 5, 6]. In particular, mathematical modelling of clonal dynamics and the emergence of resistance is critical for optimising clinical treatment strategies based on evolutionary principles [7]. The historical development of evolutionary therapies has followed a trajectory that begins with a theoretical and mathematical exploration of associated eco-evolutionary models [8, 9, 10].

Extinction therapy is a recent concept that – unlike adaptive therapy and tumour containment strategies [10, 11] – draws inspiration from the eco-evolutionary dynamics of species extinction events. The latter can be divided into two broad types [12]. Mass extinctions occur when a single impact irretrievably devastates entire populations and communities. Background extinctions are less dramatic and involve multiple events eventually leading to a species’ demise [12]. Background extinctions are much more common than mass extinctions; a single strike is often insufficient due to the presence of tolerance or resistance. In the case of evolutionary rescue, a population otherwise destined for extinction escapes through adaptive changes.

Although it is more usual to consider evolutionary rescue in a conservation context, the same theory is applicable when extinction is the goal, such as in bacterial infections or cancer [13]. Since an oncologist can control the tumour environment, they can anticipate the evolutionary trajectories of cancer clones and, in theory, follow a strategy to avoid evolutionary rescue and so cure the patient [14]. This is the principle underlying extinction therapy.

The key idea is that, even if a single strike fails to eradicate cancer cells due to resistant phenotypes, it can still render the population small and fragmented. Small populations are more vulnerable to stochastic extinction and less capable of adapting to environmental changes owing to loss of phenotypic heterogeneity [13]. Cell proliferation may also slow due to Allee effects [15]. Subsequent therapeutic strikes, if well timed, can exploit these weaknesses to initiate an extinction vortex, driving the cancer cell population below the minimum viable population threshold and hence to extinction.

Combination cancer therapies are typically designed such that cells resistant to one treatment are likely to have collateral sensitivity to another [16]. The main differences between extinction therapy and conventional combination therapy are in the timing of the second strike and the use of evolutionary principles to guide treatment. In combination or sequential therapy, the second or subsequent treatments are usually given during relapse, when the first treatment appears to have failed. Another conventional strategy is to simultaneously administer multiple drugs with collateral sensitivities from the beginning of treatment [17]. In extinction therapy, the idea is instead to attack the cancer at its weakest point, when it may well be clinically undetectable. It has been suggested that the best time to give the second strike may be while the tumour is still shrinking in response to the first therapy [18]. It follows that the success rate of extinction therapy is expected to be highly sensitive to the timing of the second strike.

There has been only one prior study of extinction therapy, which used a relatively complicated computational model to provide proof of concept [18]. Based on numerical simulations, the investigators concluded that the timing and severity of the second strike are essential determinants of extinction dynamics and that Allee effects are advantageous.

Many critical questions regarding the timing of the second strike, the time until extinction, the effect of environmental and demographic factors, and most importantly the conditions under which extinction therapy is a feasible alternative to other therapies, remain unanswered. How effective is the first strike and does it make the population vulnerable enough for further strikes to work? How do we characterise this “vulnerability”? What is the probability that a population is rescued either by pre-existing mutants or those that arise during the treatment? How do outcomes vary with the cost of resistance, density dependence, and other factors that affect clonal growth rates?

We tackle these questions in two ways. First, using ideas from evolutionary rescue theory, we develop and study the first analytical model of extinction therapy. This simple, tractable mathematical model enables us to compute extinction probabilities and to identify the optimal time for the second strike. Second, we use extensive stochastic simulations to test the robustness of our analytical results and to study the effects of additional factors. We thus establish a necessary foundation for further theoretical and experimental investigations of extinction therapy.

## 2 Methods

### 2.1 Modelling extinction therapy

Consistent with prior work [18], we study the simplest case of extinction therapy comprising only two strikes. Since further strikes can only improve treatment outcomes, we thus obtain conservative lower bounds on potential benefits. The first treatment (or strike) creates a stressful environment that we denote $E_{1}$. After switching to the second treatment, the tumour enters the second stressful environment, $E_{2}$.

Corresponding to the two treatments, we consider four cell types – sensitive to both treatments (S cells), resistant to one of the treatments but sensitive to the other $\left(R_{1}\right.$ and $R_{2}$ cells) and resistant to both treatments ($R_{1,2}$ cells). Consequently, $R_{1}$ and $R_{1,2}$ cells are resistant in $E_{1}$ and $R_{2}$ and $R_{1,2}$ cells are resistant in $E_{2}$. Even if they initially rescue the population, all $R_{1}$ cells will eventually go extinct due to the second strike. Any case of evolutionary rescue from the second strike will be due to either $R_{2}$ or $R_{1,2}$ cells.

### 2.2 Analytical methods

Our analytical modelling method is composed of two stages. First, we numerically solve a set of differential equations to obtain the population dynamics during the first treatment. Second, we use those solutions to predict extinction probability using evolutionary rescue theory.

**Figure 1:**
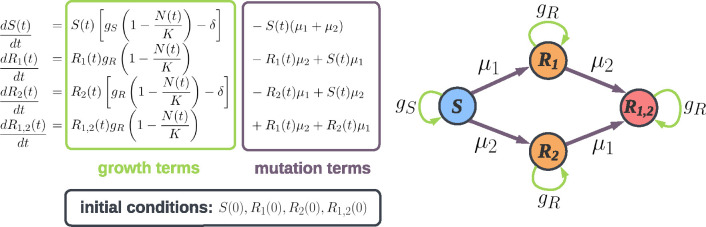
A schematic and the corresponding equations describing population growth during the first treatment (in $E_{1}$). Sensitive cells are denoted by S. Cells resistant to treatment 1(2) and sensitive to treatment 2(1) are denoted by $R_{1}\left(R_{2}\right)$. The per capita rate of acquiring resistance to treatment 1(2) is denoted by $\mu_{1}\left(\mu_{2}\right)$. Growth rates are denoted by $g_{S}$ for sensitive cells and $g_{R}$ for resistant cells, and they depend on the intrinsic birth rate, intrinsic death rate and cost of resistance (see Table 1). Initial conditions are specified by the initial population sizes of $S,\;R_{1},\;R_{2}$ and $R_{1,2}$ cells. The total initial population N(0) is the sum of these four subpopulations.

To calculate extinction probabilities with the analytical model, due to standing genetic variation and *de-novo* mutants of $R_{2}$ and $R_{1,2}$, we must obtain the population composition at the beginning of the second strike. We thus formulate the system of differential equations given in Figure 1. All system parameters and initial conditions are listed in Table 1. These equations describe logistic growth in $E_{1}$ for the four subpopulations S(t), $R_{1}(t)$, $R_{2}(t)$ and $R_{1,2}(t)$ (total population is N(t)), including the effect of mutations. Cells acquire resistance to treatment i with rate $\mu_{i}$, and the effect of treatment is captured by the per capita treatmentinduced death rate δ (assuming $\delta_{1}=\delta_{2}=\delta$). We ignore back mutations from resistant to sensitive. By solving the equations numerically, we determine the size of each subpopulation at the time of switching to the second treatment. We call this switching time τ, and the population size at this time N(τ).

Given the population composition at time τ, we first compute the probability of no evolutionary rescue due to standing genetic variation. From evolutionary rescue theory (Appendix A.1), we know that the distribution of pre-existing rescue variants can be reasonably approximated by a Poisson distribution with a rate equal to $\lambda^{SGV}=\pi_{e}\left(R_{2}(\tau )+R_{1,2}(\tau )\right)$, where $\pi_{e}$ is the probability of establishment of a single resistant lineage. The probability of establishment depends on b,d and c (see Appendix A.2 for the derivation). The probability that all pre-existing mutants go extinct in $E_{2}$ is then equal to

(1) $$P_{E}^{SGV}\left(\tau \right)=\exp\left[-\lambda^{SGV}\right]=\exp\left[-\pi_{e}\left(R_{2}\left(\tau \right)+R_{1,2}\left(\tau \right)\right)\right].$$

To find the probability that no *de-novo* rescue mutants survive in $E_{2}$, we again assume that the generation of new mutants is a Poisson process, and the number of rescue mutants in $E_{2}$ are Poisson distributed with a rate equal to

(2) $$\lambda_{2}^{DN}=\pi_{e}\mu_{2}\int_{\tau }^{t_{\text{ext}}}S\left(t\right)dt,\;\text{for}\;R_{2}\;\text{mutants},$$

(3) $$\lambda_{1,2}^{DN}=\pi_{e}\mu_{2}\int_{\tau }^{t_{ext}}R_{1}(t)dt,\;\text{for}\;R_{1,2}\;\text{mutants,}$$

where $t_{ext}$ is the time that the population goes extinct. Consequently, the probability of all *de-novo* rescue mutants in $E_{2}$ going extinct will be,

(4) $$P_{E}^{DN}(\tau )\;=exp\left[-\lambda_{2}^{DN}-\lambda_{1,2}^{DN}\right]$$

(5) $$=\exp\left[-\pi_{e}\mu_{2}\left(\int_{\tau }^{t_{\text{ext}}}S\left(t\right)dt+\int_{\tau }^{t_{\text{ext}}}R_{1}\left(t\right)dt\right)\right].$$

To compute the expression in Eq 5 numerically, we evolve the deterministic logistic growth equations for $E_{2}$, given below (Eqs 6,7). To make the computation easier, we calculate the approximate values of the integrals in Eq 5 by integrating until the population size is equal to one. In these equations, we ignore the mutations to $R_{2}$ and $R_{1,2}$, and the changes in these resistant populations, because we use only the deterministic decay of the sensitive population to calculate extinction probabilities.


(6) $$\frac{dS(t)}{dt}=S\left(t\right)\left[g_{S}\left(1-\frac{N\left(t\right)}{K}\right)-\delta_{2}\right]-S\left(t\right)\mu_{1},$$

(7) $$\frac{dR_{1}(t)}{dt}=R_{1}\left(t\right)\left[g_{R}\left(1-\frac{N\left(t\right)}{K}\right)-\delta_{2}\right]+S\left(t\right)\mu_{1}.$$

The total extinction probability as a function of τ is given by the product of $P_{E}^{SGV}$ and $P_{E}^{DN}$:

(8) $$P_{E}(\tau )\;=P_{E}^{SGV}(\tau )P_{E}^{DN}(\tau )$$

(9) $$=\exp\left[-\pi_{e}\left(R_{2}\left(\tau \right)+R_{1,2}\left(\tau \right)\right)-\pi_{e}\mu_{2}\left(\int_{\tau }^{t_{ext}}S\left(t\right)dt+\int_{\tau }^{t_{ext}}R_{1}\left(t\right)dt\right)\right].$$

With Eq 9, we study the behaviour of extinction probability as a function of τ under different conditions. We also obtain “empirical” extinction probabilities from our stochastic simulation model.

### 2.3 Stochastic simulations

Our stochastic computational model (described in Appendix A.3) specifies only the birth, death and mutation rates for all cell types in the population and outputs the extinction probability as a function of the switching point N(τ). The initial conditions and default parameter values are the same as in the analytical model. The main difference between the analytical and the stochastic simulation models is that the analytical model uses evolutionary rescue theory to calculate extinction probabilities while the stochastic simulation model uses the Gillespie algorithm to evolve the system. Each simulation must have one of three outcomes: extinction, progression, or persistence (see Table A.1). We use extinction probabilities obtained from many simulations to test our analytical predictions.

Another difference between the two models is in their birth and death rates. The analytical model is parameterised in terms of growth rates of cell lineages ($g_{S}$ and $g_{R}$), whereas the stochastic simulation model requires separate birth and death rates, accounting for the effects of competition. These effective birth and death rates used for the simulations are distinct from the intrinsic birth and death rates used in both models (see Appendix A.3 for further details). We therefore limit our analysis of demographic parameters to the analytical model.

### 2.4 A metric for comparing parameter values

In our results, we observe that $P_{E}(\tau )$ monotonically decreases as N(τ) increases. Therefore, for a given value q between 0 and 1, we obtain a corresponding value $N_{q}$, which is the maximum population size threshold that must be crossed to achieve an extinction probability greater than or equal to q. In other words, if $N(\tau )<N_{q}$, we will achieve an extinction probability of at least q:

(10) $$N_{q}=max\left\{N(\tau ):P_{E}(\tau )\geq q\right\},\;q\in \left[0,\;1\right],\tau \leq t\left(N_{min}\right).$$

This quantity can give us a measure of how fast and at what time the extinction probability drops from a high value to a low one. For instance, if the difference between $N_{0.1}$ and $N_{0.9}$ is slight, then we know that there is a sharp drop in the extinction probability at that point. We want $N_{0.9}$ to be as high as possible so that it is easier to implement the second strike. Equivalently, we want the range of N(τ) values with low $P_{E}(\tau )$ to be as small as possible. Therefore, we can say that we want the area under the curve of the $N_{q}$ vs q plot to be as large as possible. This provides us with a metric to compare different parameter values. The set of parameters for which the area under the curve is higher will result in a better outcome in terms of ease of implementation, higher extinction probabilities, or both. Furthermore, we observe a pattern of a sharp drop in extinction probabilities in a short window of $N_{q}$ values. While this pattern is not generalisable, it makes the area under the curve a reasonable choice for a metric. Alternative metrics for comparing parameter sets are described in Appendix A.4.

## 3 Results

We present results from both the numerically solved deterministic analytical model and the stochastic simulation model, and we compare the two wherever possible. Unless mentioned otherwise, we use a default set of parameters and initial conditions (see Table 1). For most of the results (all except 3.9), we take the treatment-induced death rates to be equal in both environments, i.e. $\delta_{1}=\delta_{2}=\delta$.

In practice, only the tumour size can be measured at any time, so our focus will be on the population size at the time of switching, denoted N(τ). When we compare the effect of different parameter sets and initial conditions, we will compare them at a fixed switching threshold N(τ). Since the optimal N(τ) also changes as we vary the parameters, the trend of extinction probabilities obtained at a fixed N(τ) could be different than the trend obtained at the optimal N(τ) ‘s. The rationale for using a fixed N(τ) for such comparisons is that the estimation of the optimal N(τ) may not always be possible, given that we may not know the values of all the system parameters. Therefore, it is more informative to compare treatment outcomes when switching at a fixed N(τ).

It should be noted that in the characteristic U-shaped trajectory of a population undergoing evolutionary rescue, a given population threshold for switching (N(τ)) is met twice, once before the nadir and once after. For simplicity, except where mentioned otherwise, we only consider switching points at or before the nadir (that is, prior to relapse).

### 3.1 The optimal switching time is when the population size is close to its nadir

**Figure 2:**
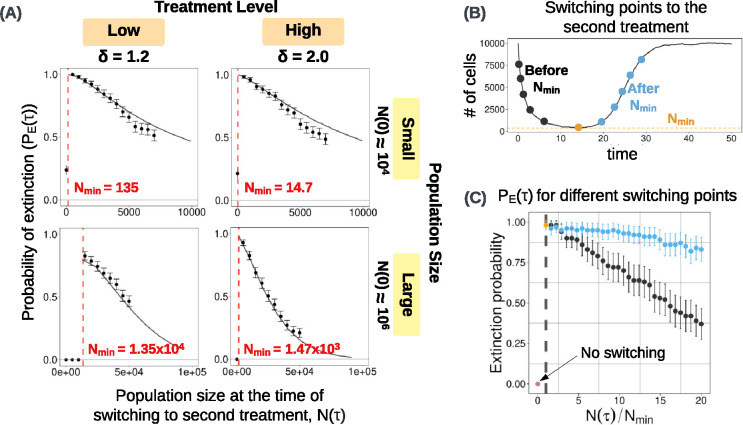
**(A)** Comparing stochastic simulation results (dots) to analytical estimates (solid black line) of extinction probabilities $P_{E}(\tau )$ for different values of N(τ) implemented before reaching $N_{min}$ (on the x-axis). Results for two population sizes, two treatment levels each, are shown. The expected $N_{min}$ is shown in red (calculated with the analytical model). For all cases, $P_{E}(\tau )$ is maximised near $N_{min}$, and monotonically decreases as N(τ) is increased further. Extinction probabilities are computed using the outcomes of 500 simulations. Error bars show 95% binomial proportion confidence intervals. **(B)** An illustration of the points of switching, before and after $N_{min}$, implemented with the same random seed. See Appendix A.3 for a description of the algorithm for these simulations. The simulation results are shown in Figure **(C)**, where the black points indicate extinction probabilities when the points of switching are before the $N_{min}$ and blue points indicate N(τ)’s implemented after $N_{min}$. The yellow point denotes extinction probability at $N_{min}$, which is the highest. Extinction probability without extinction therapy (no switching) is shown in pink and corresponds to N(τ)=0. Extinction probabilities are calculated by considering the outcomes of 100 sets of simulations with different random seeds. Since the $N_{min}$ is different for each random seed, we take the average over the 100 random seeds to obtain the mean $N_{min}=1.47\times 10^{3}$. All parameters are set to their default values. Similar plots for different treatment values are shown in Figure A.2. Error bars show 95% binomial proportion confidence intervals.

Our analytical and stochastic models both show that the optimal N(τ), in terms of maximising extinction probability, is close to the minimum population size reached in the absence of a second strike (Figure 2). We call this nadir $N_{min}$, and it can be calculated by numerically solving the system of differential equations shown in Figure 1.

To explain why the optimal N(τ) is close to $N_{min}$, we refer to Eq 9 and see that the maximum $P_{E}(\tau )$ will be achieved when the sum of all Poisson rates of generation (the λ’s) of rescue mutants is minimised. The integral terms are minimised close to $N_{min}$. Moreover, in the terms constituting $\lambda^{SGV}$, the decay in the $R_{2}$ population dominates over the increase in population size due to mutations and growth of $R_{1,2}$ cells. Hence the rescue population keeps decreasing as we move towards $N_{min}$.

In Figure 2(A), we see that the stochastic simulation results match well with the analytical predictions. For the large population size $\left(N(0)\approx 10^{6}\right)$, there are very few significant deviations from the prediction. For the smaller population size $\left(N(0)\approx 10^{4}\right)$, observations close to $N_{min}$ match the analytical predictions, but there is a deviation as we move towards higher values of N(τ). Since the resistant subpopulations are very small initially and require some time to establish in the population, predictions from the deterministic analytical model may not accurately describe subpopulation growth at the beginning of the first strike. Thus, for large N(τ) (that is, for switching times closer to the beginning of the therapy), the stochastic simulation results deviate slightly from the analytical predictions. This effect is not seen in the larger population perhaps because the $P_{E}(\tau )$ is very low for switching points close to the initial population size. This drop can be seen in Figure 2(A) (bottom row), where the expected extinction probability is close to 0 even for values as small as $N(\tau )=10^{5}$, which is only 10% of the initial population size.

### 3.2 It is better to implement the second strike after the nadir than before

If only the first treatment is given then, due to the establishment of resistant variants, relapse is inevitable. This is why we obtain an $N_{min}$ in the absence of a second strike. In our prior simulations and analytical results, we have considered only switching before $N_{min}$ is reached. Due to relapse, the same switching points can also be selected after $N_{min}$. This raises the question of whether it is better to treat before or after the nadir.

We used our stochastic simulation model to address this question, comparing treatment outcomes for multiples of $N_{min}$ ranging from $N_{min}$ to $20N_{min}$, as shown in Figure 2(C) (see Appendix A.3 for further details of the algorithm). These additional simulation results confirm that the maximum $P_{E}(\tau )$ is obtained near $N_{min}$. We also see that switching points after $N_{min}$ (Figure 2(C), blue points), have significantly higher extinction probabilities than those before $N_{min}$ (Figure 2(C), black points). This result holds for a range of treatment levels (Figure A.2). Therefore, when the time at which the tumour population reaches $N_{min}$ cannot be determined precisely, it is generally better to wait until after the nadir than to switch to the second treatment too early. The treatment is likely to result in a significantly higher extinction probability if the same switching point threshold is implemented after crossing the nadir.

We hypothesize that this result is due to two effects working in the same direction. First, since the ratio of $R_{1}$ cells keeps increasing before the second strike, the cost of resistance results in an overall higher death rate than would be observed at the same population size before $N_{min}$. Second, the pre-existing $R_{2}$ population decays to a smaller size as we wait longer, which results in a smaller rescue population during the second treatment. Consequently, the so-called “window of opportunity” extends further to the right of the nadir than to the left. In fact, for a given N(τ), the extinction probability to the right of the nadir can be as much as twice that on the left.

### 3.3 Two-strike extinction therapy is feasible only in small tumours

**Figure 3:**
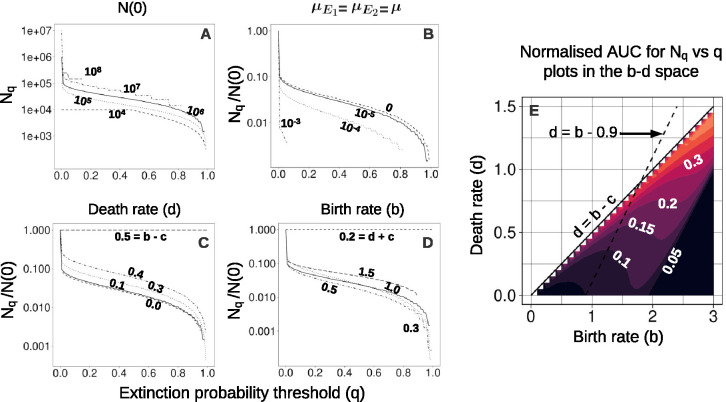
Effects of varying parameter values or initial conditions. In **A-D**, the title of each plot indicates the parameter or initial condition that varies between curves, and the solid curve corresponds to the default value (Table 1). (A) $N_{q}$ versus q for different initial population sizes, varied from 10^4^ to 10^8^. Larger initial population sizes do not allow extinction probabilities higher than 0.1. **(B)** $N_{q}$ versus q for different mutation rates. **(C)** $N_{q}$ versus q for different death rates. For death rates higher than 0.5, resistant cells have a negative growth rate (because b−c=0.5). **(D)** $N_{q}$ versus q for different S cell intrinsic birth rates. For birth rates less than 0.2, the resistant cells have a negative growth rate. **(E)** Heatmap of the normalised area under the curve (AUC) of the $N_{q}$ vs q plots for different parameter values in b−d space. Only non-negative growth rates (excluding the effects of treatment) are considered (d≤b−c, solid black line). The dashed black line indicates the set of birth and death rates corresponding to our default growth rate $\left(b-d=g_{S}=0.9\right)$.

Using the analytical model, we compare extinction probabilities at different values of $N_{q}$ (not normalised) for different initial population sizes N(0), bearing in mind that the resistant population size scales with N(0). We observe that the absolute values of $N_{q}$ for q close to 1 do not vary by more than an order of magnitude when N(0) ranges over three orders of magnitude, from 10^4^ to 10^7^ cells (Figure 3A, Table A.2). This implies that, within this range of initial tumour sizes, a high extinction probability can be achieved by applying the second strike at a sufficiently small population size (mostly determined by the treatment dose, growth rates, and other parameters). Nevertheless, if N(0) is larger than 10^8^ cells then the extinction probability never exceeds 0.1 (Figure 3A). There is therefore a limit on the size of tumours for which two-strike extinction therapy is likely to succeed.

### 3.4 Mutation during treatment reduces the extinction probability

Next, we examine how ongoing mutation influences the treatment outcome. In our model, there are four types of mutation (Figure 1) and the total mutation rate is $2\left(\mu_{1}+\mu_{2}\right)$. In Figure 3B, we see that increasing the total mutation rates in both $E_{1}$ and $E_{2}$, while keeping N(τ) and the initial frequency of resistance unchanged, results in lower extinction probability. We observe the same trend if we change the mutation rate in only one environment (Figure A.3). This effect is due to higher mutation rates resulting in a larger rescue population size, and hence a higher probability of evolutionary rescue. For a total mutation rate as high as 10^−3^, the extinction probability never exceeds 0.1. On the other hand, the benefit of decreasing the mutation rate greatly diminishes after $\mu =10^{-5}$, as the number of pre-existing $R_{2}$ mutants becomes the dominant factor, which sets an upper bound on the extinction probability when we switch before reaching $N_{min}$. In the extreme, unrealistic case of abundant pre-existing resistance and very low mutation rates, the optimal switching point would be long after $N_{min}$, when the $R_{2}$ population has fallen to close to zero.

### 3.5 Extinction probability increases with death rate and turnover

**Figure A.1:**
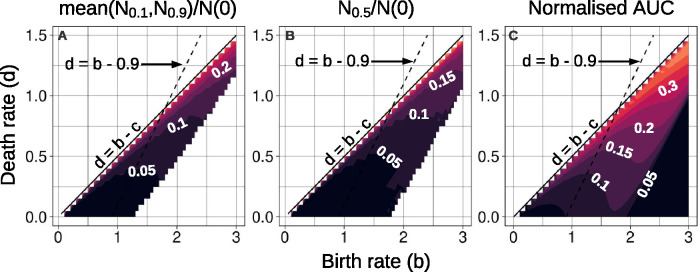
Comparing treatment outcomes for different parameter values in the b−d space using three different metrics. For each metric, values of birth and death rates are chosen such that the resistant cells have a positive growth rate. The dashed line indicates the set of birth and death rates which result in a constant intrinsic growth rate (equal to the default value of $g_{S}=0.9$. **(A)** The metric is defined as the normalised mean of $N_{0.1}$ and $N_{0.9}$. The bottom right region gives invalid values using this metric because $N_{0.9}$ is not defined for those parameters. This is because the extinction probabilities in that region are lower than 0.9. **(B)** The metric is defined as the normalised $N_{0.5}$. Similar to the metric used in plot A, the parameters in the bottom right region give extinction probabilities less than 0.5. **(C)** The metric we use for our analysis in the main text is the normalised area under the curve of the $N_{q}$ vs q plot for parameters in the b−d space. This metric is favoured because it gives smooth curves on the plot, which also does not contain an invalid region.

**Figure A.2:**
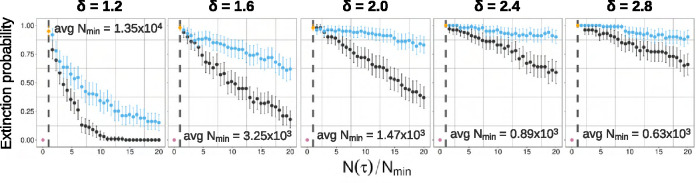
Simulation results for points of switching before and after $N_{min}$. For the same random seed, extinction probabilities for different switching points relative to $N_{min}$ are calculated (see Appendix A.3). The black points indicate extinction probabilities when the points of switching are before the $N_{min}$ and blue points indicate N(τ)’s implemented after $N_{min}$. The yellow point denotes extinction probability at $N_{min}$, which is the highest. Extinction probability without extinction therapy (no switching) is shown in pink and corresponds to N(τ)=0. See Figure 2(B) for the legend. All parameters are set to their default values, except the treatment level, which is indicated on top of each panel. Error bars show 95% binomial proportion confidence intervals. All extinction probabilities are obtained by considering the outcomes of 100 runs of the simulation with the different random seeds. The average $N_{min}$ over all the simulations is also shown for each panel.

**Figure A.3:**
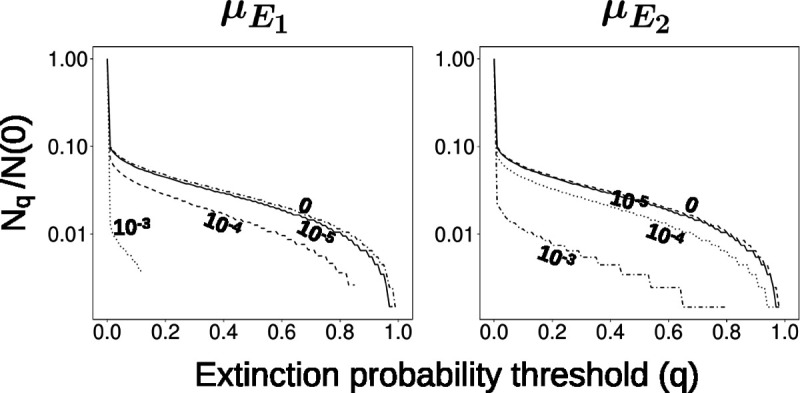
Normalised $N_{q}$ vs q plot for different values of total mutation rates in $E_{1}$ and $E_{2}$. The value of $\mu_{E_{1/2}}$ is kept constant at 10^−5^ when $\mu_{E_{2/1}}$ is varied (right/left). Changing the total mutation rate in both environments individually has the same effect (qualitatively). A higher mutation rate results in lower extinction probabilities. This figure is obtained using the analytical model only.

To compare treatment outcomes in b−d space, we plot the normalised area under the curve (AUC) for the $N_{q}$ versus q plots for feasible combinations of birth and death rates (Figure 3E). We observe that in the lower right region (high birth rates, low death rates), the AUC is very low. This result also holds for alternative metrics (Figure A.1 in Appendix A.4). This leaves us with a diagonal band in b−d space within which it is possible to attain high extinction probabilities.

Within this “good” region, we make three major observations. First, a higher death rate results in a higher extinction probability. Second, as the birth rate increases, extinction probability first decreases and then increases. Third, we observe that the extinction probability increases as we increase the turnover (defined as the sum b+d) while keeping the intrinsic growth rate $g_{S}$ constant (dashed line in Figure 3E). Note that the cost of resistance is always a fixed fraction of the intrinsic birth rate (birth rate of S cells). It follows that when increasing turnover while keeping the growth rate $g_{S}$ constant, the growth rate $g_{R}$ of resistant cells decreases. This leads to a smaller rescue population, contributing to the increase in extinction probability.

Another effect of turnover may relate to the establishment probability of resistant mutants. As noted in Appendix A.2, turnover appears in the expression for estimating the establishment probability $\pi_{e}$. Higher turnover leads to a lower $\pi_{e}$. If it is harder for resistant lineages to establish then there will be fewer rescue lineages, leading to better treatment outcomes.

### 3.6 A cost of resistance is beneficial but not essential

**Figure 4:**
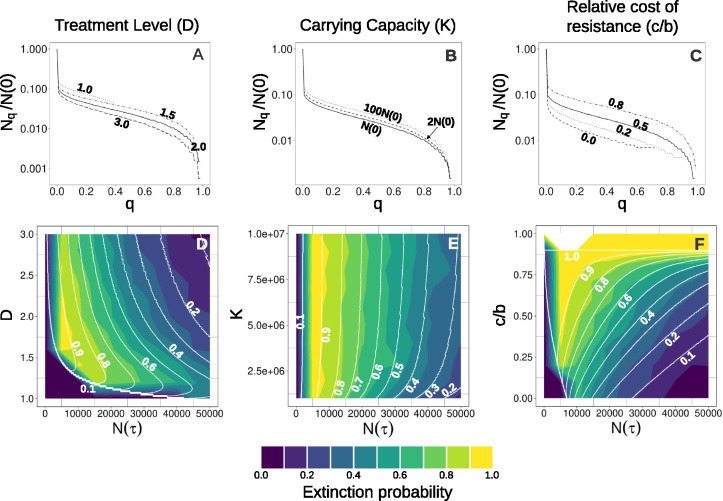
Normalised $N_{q}$ vs q plots (top row) and extinction probability heatmaps (bottom row) for three system parameters. In all the heatmaps, the solid white contours depict analytical results. Stochastic simulation results are denoted by the colour scale. Extinction probabilities from the stochastic model are obtained by using the outcomes of 500 simulations with the same parameter values and initial conditions. The top row shows only analytical results. The solid line in each plot in the top row indicates the curve for default parameter values and initial conditions (see Table 1). **(A,D)** Treatment levels for both environments are altered together $\left(\delta_{1}=\delta_{2}=\delta )\right.$. The default treatment level is δ=2.0 per unit of time. This particular range of treatment levels is taken because 0.9 is the intrinsic growth rate of the sensitive cells, due to which δ<0.9 will only give positive growth rates for all cells in the population. The bottom left region of the plot has very low extinction probabilities because $N(\tau )<N_{\text{min}}$ at those points. **(B,E)** Carrying capacity for the system is varied. The default carrying capacity is equal to the default initial population size. **(C,F)** The cost of resistance (relative to the birth rate of S cells, b) is altered. The default value of the cost is c=b/2=0.5.

Next, we examine the effect of varying the cost of resistance (c), which is the difference between the growth rates of S cells and resistant cells ($R_{1},\;R_{2}$ and $R_{1,2}$). As the cost of resistance increases, relative to the intrinsic birth rate, the size of the rescue population decreases, leading to a higher extinction probability (Figures 4C,F). Nevertheless, for costs of resistance as low as 20% of the birth rate, it is still possible to obtain extinction probabilities as high as 0.8. Even if there is no cost of resistance, we obtain moderately high extinction rates of up to 0.6 in simulations (Figure 4F).

### 3.7 Intermediate doses maximise extinction probability

To examine dose effects, we simulate a range of treatment levels for each of several values of N(τ) (the total population size at the time of switching to the second treatment) while keeping the first and second treatment doses equal. Surprisingly, we observe that for each N(τ) there is an optimum dose, above which the extinction probability decreases (Figure 4A and 4D).

This somewhat counter-intuitive result is explained by the interaction of several factors. For a given N(τ), a lower dose during $E_{1}$ leads to a higher $R_{1}$ population at the time of switching. This is beneficial because, due to the cost of resistance, the $R_{1}$ population decays faster than the sensitive population during $E_{2}$. A lower dose during $E_{1}$ also gives more time for the preexisting $R_{2}$ population to decay. On the other hand, a lower dose gives more time to generate new rescue mutants during both $E_{1}$ and $E_{2}$. What we observe is that the beneficial effects of a lower dose outweigh the detrimental ones.

**Figure A.4:**
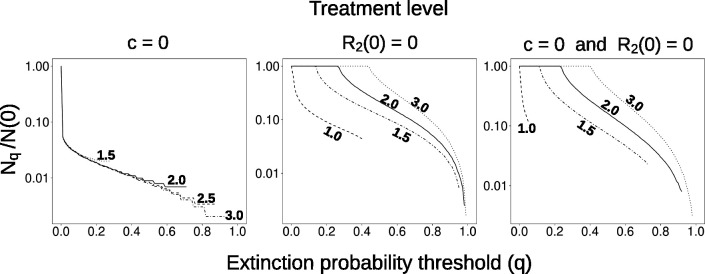
Normalised $N_{q}$ vs q plot for different treatment levels under three conditions – no cost of resistance, no initial $R_{2}$ population, and the combination of the two. The presence of either of the two factors, cost of resistance and initial $R_{2}$ population results in an increase in $P_{E}(\tau )$, while the absence of both factors reveals the effect of altering treatment level. This figure is obtained using the analytical model only.

To verify this intuition, we indirectly eliminate each of the two factors favouring a lower dose by setting the cost of resistance and the initial $R_{2}$ population to zero (see Figure A.4). In the default case, when both effects are present, we observe that a higher treatment level leads to a lower $P_{E}(\tau )$. In the absence of both factors, we obtain the intuitive result of an increase in $P_{E}(\tau )$ due to higher treatment levels. This is because a higher treatment level means a higher rate of decay and less probability of generation of rescue mutants in the second phase.

### 3.8 Extinction probability is insensitive to carrying capacity

As the carrying capacity is increased from N(0) (default value), we see a slight increase in extinction probability, but this effect saturates before K=10N(0). This is demonstrated in Figures 4B using the analytical model and in Figure 4E with stochastic simulation results.

**Figure A.5:**
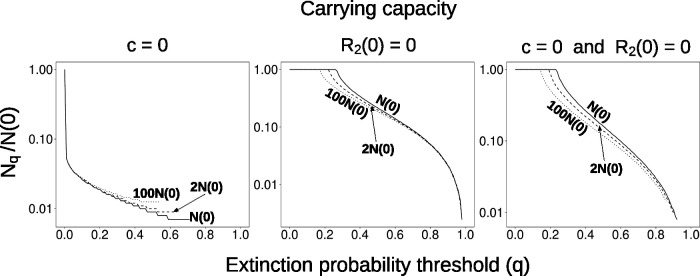
Normalised $N_{q}$ vs q plot for different values of the carrying capacity under three conditions – no cost of resistance, no initial $R_{2}$ population, and the combination of the two. The presence of either of the two factors, cost of resistance and initial $R_{2}$ population results in an increase in $P_{E}(\tau )$, while the absence of both factors reveals the effect of changing the carrying capacity. This figure is obtained using the analytical model only.

Systems with a lower K have an extra constraint on population growth since the initial population is closer to the carrying capacity. In our model this results in a lower decay rate for S cells and a higher growth rate for $R_{1}$ cells. As explained in Section 3.7, the $R_{1}$ and $R_{2}$ population sizes at a given N(τ) affect the extinction probability. By default, if both factors are present then an increase in K causes an increase in $P_{E}(\tau )$. The individual effects of both these factors are shown in Figure A.5. The same figure shows that, in the absence of both factors, an increase in the carrying capacity results in a small decrease in $P_{E}(\tau )$.

### 3.9 A low first treatment dose followed by a high second treatment dose maximises extinction probability for a fixed N(τ)

Finally, we challenge our assumption of equal treatment doses during the two phases of extinction therapy $\left(\delta_{1}=\delta_{2}=\delta \right)$. Here we mainly compare different pairwise combinations of “high” and “low” doses in the two environments. The “low” treatment level is close to the intrinsic growth rate of the population (equal to 0.9 by default). The default treatment level of δ=2 is considered “high”.

**Figure 5:**
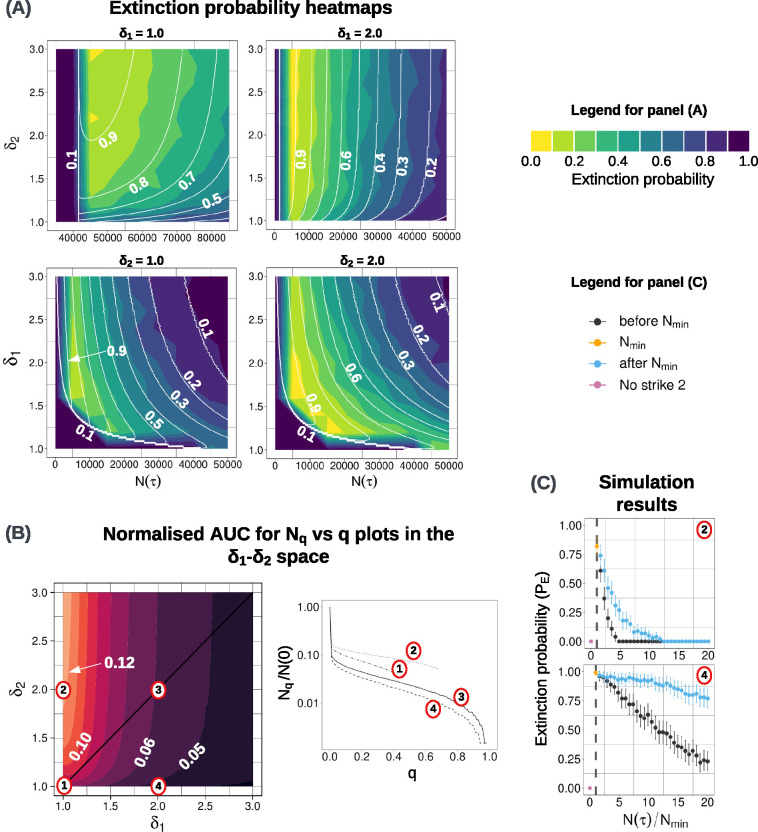
**(A)** Extinction probability heatmaps for four cases, each with one of the treatment levels at a constant high or low value. Plots in the top(bottom) row are obtained by varying $\delta_{2}\left(\delta_{1}\right)$ while keeping $\delta_{1}\left(\delta_{2}\right)$ constant. Solid white lines indicate analytical predictions of extinction probability while the colour scale represents simulation results. Extinction probabilities from the simulations are calculated by using 500 runs with the same parameter values and initial conditions. Plots in the bottom row show a similar behaviour to Figure 4D. **(B)** The heatmap of normalised AUC for $N_{q}$ vs q plots of points in the $\delta_{1}-\delta_{2}$ space (left) and the normalised $N_{q}$ versus q plots for four points (right). These are obtained solely from the analytical model. **(C)** Simulation results for two points with unequal treatment levels in the two treatment phases. As in Figure 2(C), switching points before and after the crossing of $N_{min}$ are considered with the same random seed. Extinction probabilities are obtained from 100 paired simulations with different random seeds.

Figure 5(A) shows extinction probability heatmaps for four cases, each with one of the treatment doses kept high or low. In the top-left panel in the figure, where $\delta_{1}$ is kept low, we observe that the range of N(τ) values (on the x-axis) starts at a much higher value. This is because the $N_{min}$ for this set of parameters is much larger than $N_{min}$ in most other cases, where $\delta_{1}$ is not close to the intrinsic growth rate. Furthermore, we observe that the region of high $P_{E}(\tau )$ (say, > 0.8) is also larger than in other cases. Together, both these factors tell us why a low $\delta_{1}$ value is beneficial. A relatively large region of high $P_{E}(\tau )$, starting at a relatively high N(τ) makes it easier to implement extinction therapy. In contrast, the bottom two plots in the panel show a behaviour similar to the default case (Figure 4D). However, high values of $\delta_{2}$ result in larger regions of high extinction probability.

All these observations are corroborated by Figure 5(B), which shows us a condensed overall picture of how the treatment outcome varies in $\delta_{1}-\delta_{2}$ space. Again, we observe that the highest normalised AUC is obtained when $\delta_{1}$ is low and $\delta_{2}$ is high. A normalised $N_{q}$ versus q plot (Figure 5(B), right) for four points in $\delta_{1}-\delta_{2}$ space confirms that the point in the low $\delta_{1}$-high $\delta_{2}$ range produces the best treatment outcome because it gives a higher extinction probability at the same N(τ).

In Figure 5(C), we compare two cases with unequal doses in the two environments. As observed in Figure 2, it is better to switch to the second treatment after the $N_{min}$ nadir. However, in the case corresponding to point 2 $\left(\delta_{1}=1,\delta_{2}=2\right)$, we see comparatively low extinction probabilities even though it is the better parameter set as determined by panels (A) and (B). This is because the N(τ) ‘s in both the plots in Figure 5(C) are not the same. Since the x-axis is relative to $N_{min}$, we cannot directly compare the two parameter sets. What we can conclude is that the result in Section 3.2 holds even when the treatment doses are unequal.

## 4 Discussion

Extinction therapy is a novel evolutionary therapy that aims to push tumours to extinction by exploiting stochasticity in small and vulnerable populations. This is done by applying multiple “strikes” or treatments at appropriate times. A tumour that responds well to the primary therapy is primed for a second strike when it is small and susceptible to stochastic effects. Our aim then is to “kick it while it’s down” [14].

Here we have developed the first analytical model of extinction therapy, which being mathematically tractable yields clearer explanations and more general results than previous approaches [18]. We have also developed a complementary stochastic simulation model, which generally confirms the accuracy of our analytical predictions. We have sought to make both models as simple as possible, with minimal assumptions about parameter values and relations between different quantities.

We have used these new mathematical and computational models to investigate the optimal timing of the second strike and how the treatment outcome depends on crucial system parameters including treatment levels, mutation rate, and growth rates. The combination of analytical and computational analyses, both derived from the principles of evolutionary rescue, arms us with powerful tools to explore extinction therapy in a wide range of scenarios, with a solid basis in eco-evolutionary theory.

### When do we get the best treatment outcome?

The optimal second strike threshold (optimal N(τ)) is one of the most important quantities to determine when studying extinction therapy. The ability to analytically predict this optimal switching point for a large range of parameter values promises to aid the design of effective treatment schedules for extinction therapy. By numerically solving our analytical model (Section 3.1), we have shown that the optimal N(τ) is approximately equal to the minimum population size $\left(N_{min}\right)$ that would be reached in the absence of a second strike. This result – which is supported by extensive simulations (Figures 2(C) and A.2) – is consistent with a hypothesis proposed in the previous investigation of extinction therapy [18].

Previous research did not consider post- $N_{min}$ switching points and assumed that the pre- $N_{min}$ thresholds are better in terms of treatment efficacy. On the contrary, we have shown that switching slightly after the $N_{min}$ nadir results in a significantly higher extinction rate than slightly before (Section 3.2). Since it is unreasonable to expect switching to the second strike exactly at the optimal point, we conclude that it is better to wait a bit longer and risk missing the optimal N(τ) than to apply the second strike too early. However, one should certainly not wait until the tumour becomes detectable again (as is the current practice) because that negates the benefit of exploiting the vulnerability of small populations.

### What factors determine the success of extinction therapy?

Our systematic exploration of the model parameter space reveals several noteworthy effects on treatment outcomes. First, two-strike extinction therapy is likely to succeed only in relatively small tumours. Below this threshold, the initial population size has little effect on the range of switching points that give high extinction probabilities (Section 3.3), assuming that these switching points can be attained with the first treatment. Second, mutation during either treatment is detrimental for extinction therapy (Section 3.4). This result suggests, for example, that mutagenic therapies may be less appropriate. Third, we find that higher death rates and higher turnover are beneficial to extinction therapy, as has previously been shown for adaptive therapy [19]. Fourth, although a high cost of resistance is predictably beneficial, we find that extinction therapy can outperform conventional treatment even when this cost is small or non-existent. Therefore, in common with adaptive therapy [11], extinction therapy is not contingent on a cost of resistance. Sixth, although a higher carrying capacity allows more tumour growth, we found that changes in carrying capacities have little effect on treatment outcome (Section 3.8). Understanding the effects of carrying capacity will be especially important when interpreting experimental tests of extinction therapy.

### What are the optimal doses?

The treatment levels during the two strikes ($\delta_{1}$ and $\delta_{2}$) are the easiest model parameters to control in practice. Surprisingly, at least with a large cost of resistance and pre-existing mutants, we found that a generally more aggressive approach results in lower extinction probabilities for a given N(τ) (Section 3.7). This result emphasizes the importance of timing in extinction therapy – a stronger treatment with a poorly chosen switching time is worse than a weaker treatment given at the right time. In the more general case of unequal doses $\left(\delta_{1}\neq \delta_{2}\right)$, we found that the best treatment outcome is obtained when the treatment level of the first strike is low (close to the intrinsic growth rate of the population) and the second strike dose is high (Section 3.9). In this case, the optimal switching threshold is relatively high, which may be easier to achieve in practice. An interesting implication of this result is that the two treatments need not both be very effective. Although we find that a high $\delta_{2}$ is optimal, our results are consistent with a prior hypothesis [18] that extinction therapy is a viable option even if the second strike is not very strong.

### What are the limitations of our study?

Our results are subject to certain methodological assumptions and limitations. First, because our analytical modelling method neglects stochastic effects during the first treatment, our analytical predictions are less accurate in cases when these effects are influential, such as for large N(τ) in small populations. However, for reasonably large initial tumour sizes, our analytical predictions closely match stochastic simulation results, indicating that our method is appropriate for most relevant scenarios (Figure 2(A)). Second, although we have used simple models with minimal assumptions to ensure that our main findings are qualitatively robust, we have not explored all plausible functional forms. For example, the effect of changing the mutation rate might be different in a model in which mutations occur only at the time of cell division. Third, whereas we have examined the effects of varying parameters for general fixed N(τ) values, we might observe different trends were we to assume that switching always occurs at the optimal N(τ) (which changes with the parameter values). Fourth, because we have considered relatively small tumours, our results are most relevant to metastases or to residual tumour. Nevertheless, we expect that further strikes, following the same principle, would lead to higher extinction probabilities for larger tumours, making extinction therapy viable in a wider range of scenarios.

### When should extinction therapy be used?

Extinction therapy holds most promise as an alternative to conventional therapy in cases where a very good initial response to treatment is typically followed by relapse. Proposed targets include locally advanced rectal adenocarcinoma [20], metastatic prostate cancer [14], and paediatric sarcomas [21]. It may also be a wise strategy when one of two available treatments is less effective than the other. Conversely, if resistant cells are abundant and have relatively high fitness then extinction therapy is unlikely to succeed and a long-term tumour control strategy such as adaptive therapy could be a better option [10, 11]. Even when it may be theoretically optimal, extinction therapy crucially depends on the availability of effective treatments with low cross-resistance, and methods for monitoring tumour burden over time [21].

### Conclusion and future directions:

We have shown that extinction therapy is a theoretically sound concept that, in certain scenarios, could plausibly increase cancer cure rates. Our work provides a necessary foundation for further mathematical investigation and justification for experimental testing of this innovative strategy. An important topic for further mathematical analysis is extinction therapy with more than two strikes. Previous work on the optimal scheduling of multiple treatments [22, 23, 24] suggests that alternating two treatments is a theoretically sound approach. An alternative strategy, more in line with the original conception of extinction therapy, is to switch to a new treatment whenever possible. Other immediate directions for mathematical investigation include accounting for cross-resistance and considering alternative biological assumptions, such as modelling resistance as a continuous, plastic trait.

## Figures and Tables

**Table 1:** List of parameters and initial conditions used in the analytical and stochastic simulation models, along with their default values.

Symbol	Description	Default value

K	Carrying capacity of the system	N(0)
b	Per capita birth rate of S cells	1.0
d	Per capita death rate of all cell types	0.1
c	Cost of resistance	0.5
$\mu_{1}$, $\mu_{2}$	Mutation rate for acquiring resistance to treatment 1, 2	2.5 × 10^−6^
$\delta_{1}$, $\delta_{1}$	Per capita death rate due to treatment 1,2	2.0

S(0)	Initial population of S cells	10^6^
$R_{1}(0)$	Initial population of $R_{1}$ cells	100
$R_{2}(0)$	Initial population of $R_{2}$ cells	100
$R_{1,2}(0)$	Initial population of $R_{1,2}$ cells	0

Note that for the analytical mode, we use the values of growth rates for sensitive and resistant cells, gs=b−d and $g_{R}=b-c-d$ respectively.

## A Appendices

### A.1 Evolutionary Rescue Theory

The phenomenon of the prevention of extinction due to adaptive evolutionary changes is termed evolutionary rescue. It is different from other forms of rescue like demographic rescue which occurs when the population is rescued due to population dispersal and immigration of fitter phenotypes. In evolutionary rescue, resistance must emerge at a timescale similar to that of population decay under environmental stress, which is why it is often described as a “race against extinction” [25]. While the conventional goal of evolutionary rescue is to maximise the probability of rescue, the same mathematical formulations can be applied with the aim of extinction. For our deterministic analytical model, we use the theory of evolutionary rescue (ER) to find the probability of extinction in cancer populations undergoing extinction therapy. Mathematical models of ER analytically study systems that experience external stress, determining how and under what conditions is it possible for the population to undergo evolutionary changes in order to rescue the population [26]. There are many factors that affect this process, broadly divided into genetic factors, demographic factors and external factors [25]. Depending on the system one is working with, different factors end up playing important roles.

In our case, a mathematical model of evolutionary rescue in an isolated, asexual population under two different environmental stresses, we must have three essential components corresponding to the interactions between the three most important determinants of population extinction [13, 27, 28]. Small population size,low genetic variance and a high degree of environmental stress are factors that might lead to species extinction [26]. Small populations are at a higher risk of stochastic extinction. Furthermore, the total mutation rate is lower because the number of individuals is small, so the generation of resistant lineages is slow. Even if resistant lineages exist, they might die out due to demographic stochasticity. The risk is even greater if one considers Allee effects (reduced growth rate at low population sizes) [15]. Therefore, changes in population size with environmental stress must be specified.

Prior to the onset of stress, it is assumed that the resistant variants are rare or non-existent. If they are abundant, it is almost certain that the population will survive [29]. Given these conditions, one can assume density-independent growth for the resistant mutants in the beginning and use branching processes to model it [30]. The growth model we use is described in Appendix A.2.

A population is rescued from extinction when one or more resistant lineages are fixed in the population. The generation of resistant mutants is controlled by the mutation rate, which may or may not depend on the degree of stress. The mutants that ultimately establish themselves in the population and lead to evolutionary rescue are called rescue mutants. The probability of establishment of a resistant mutant can be approximated by Haldane’s 2s [31], where s is the selective advantage of the mutant over the wild-type, which depends on the degree of stress. For a more general, continuous-time estimate, we use diffusion approximation to get the establishment probabilities of resistant lineages starting with a single cell [32, 13],

(11)
$$\pi_{e}=1-exp\left[\frac{-2(b-d)}{\left(b+d\right)}\right]$$

This equation is derived in Appendix A.2 and used in Section 2.2.

An important distinction is that the probability of rescue by pre-existing mutants (from before the onset of stress, called standing genetic variation) is different from that of new mutants (via de-novo mutations) [30]. This is because the pre-existing mutant (SGV) lineages typically get more time to grow than the de-novo mutants (DN). There are several expressions by different authors [27, 33, 30] for the probability of evolutionary rescue by both these classes of mutants, but considering the common conceptual basis, they all reduce to the following form:

(12)
$$P_{ER}^{SGV}=1-exp\left[-N(0)\pi_{e}f(0)\right]$$

(13)
$$P_{ER}^{DN}\;=1-exp\left[-\mu \pi_{e}\int_{0}^{t_{ext}}N(t)dt\right]$$

where N(0) is the population size at the onset of stress (t=0), μ is the total per capita, per unit time mutation rate after the onset of stress, f(0) is the frequency of resistant variants at t=0 and $t_{ext}$ is the time at which the population would go extinct if it is not rescued. The above equations are derived on the basis of one main assumption — the generation of rescue mutants in the population can be approximated by a Poisson process [13]. Once the rate of the Poisson process is calculated, it is easy to obtain the probability of extinction which will be equal to the probability that no rescue mutants are generated.

Additionally, we make assumptions about the density independence of rescue lineages and that the probability of establishment of a resistant mutant is constant throughout. There is extensive literature on what happens when these assumptions do not hold. Analytical results can be derived in all such situations using stochastic methods and taking continuous time approximations [33, 27, 34].

To summarise, with evolutionary rescue models, one can derive the probability of extinction of a population under stress, given its initial state. In Section 2.2, we use these results extensively in the context of extinction therapy. The principles of evolutionary rescue provide a foundation for building the theoretical formulation of extinction therapy. There are, of course, significant differences to account for — firstly, most evolutionary rescue models consider either one abrupt change in the environment or a continuous, gradual change [28]. However, extinction therapy is based on using two or more subsequent strikes, all of which are abrupt changes in the environment. Second, most existing models consider a single resistant variant (an exception is G. Martin et al. (2013) [27]), while the existing model for extinction therapy [18] works with a continuum of resistance effects. We choose to develop the simpler case in which we have discrete genetic variation (two resistant variants). We therefore theoretically understand extinction therapy as the prevention of evolutionary rescue.

### A.2 Derivation of $\pi_{e}$

In this section, we provide the derivation of the establishment probability of a single lineage of resistant cells. This result is taken from Lambert, 2006 [32]. As described in Appendix A.1, the establishment probability is the probability with which resistant mutants establish themselves in the population and lead to evolutionary rescue. These mutants are then called rescue mutants.

We use the CB process, a continuous time, real-valued branching process to model population dynamics of the resistant lineages. We chose this process because CB processes are the only diffusion processes that satisfy the additive property of the well-known BGW processes, which are commonly used to model stochastic population dynamics. Therefore, a CB process can be used to model the total population size summed over several lineages evolving independently. In our case, we use a CB process which is a continuous function of time, also called branching diffusion. As described in Lamperti, 1967 [35], branching diffusions are strong solutions of SDEs of the form:

(14)
$$dZ_{t}=rZ_{t}dt+\sqrt{\sigma Z_{t}}dB_{t},$$

where B is the standard Brownian motion, r∈R is the intrinsic growth rate, and $\sigma \in R^{+}$ is the reproduction variance, defined as [27]:

(15)
$$r\;=\underset{\Delta t\to 0}{lim}\;\frac{E\left(\Delta Z_{t}|Z_{t}\right)}{\Delta tZ_{t}}$$

(16)
$$\sigma =\underset{\Delta t\to 0}{lim}\;\frac{Var\left(\Delta Z_{t}|Z_{t}\right)}{\Delta tZ_{t}}$$

With this equation, we use a general result from diffusion theory that the probability u of the diffusion hitting an absorbing barrier $z_{0}$ solves the equation Gu(z)=0, where z is the initial condition $\left(Z_{0}=z\right)$. Here, G is the infinitesimal generator of the diffusion and characterizes the behaviour of the diffusion at small time intervals. For our one-dimensional diffusion (Equation 14), G is of the form,

(17)
$$Gf(z)=rf^{'}(z)+\frac{\sigma }{2}f^{''}(z)$$

For the absorbing barrier $z_{0}=0$, we will obtain the extinction probability of a resistant population starting with z cells by solving Gu(z)=0, with boundary conditions u(0)=1 and u(∞)=0. The solution of this differential equation is,

(18)
$$u(z)=exp\left[\frac{-2rx}{\sigma }\right]$$

As explained in Martin et al., 2013 [27], the reproduction variance for a simple birth-death process with the birth rate b and death rate d can be approximated by b+d (turnover). Consequently, we obtain the following expression for the establishment probability, defined as one minus the extinction probability, for a resistant lineage starting from a single cell.

(19)
$$\pi_{e}=1-exp\left[\frac{-2(b-d)}{\left(b+d\right)}\right]$$

We use this result to compute the extinction probabilities with our deterministic analytical model in Section 2.2.

### A.3 Stochastic Simulation Model

The total population at time t is $N(t)=S(t)+R_{1}(t)+R_{2}(t)+R_{1,2}(t)$, as defined in Section 2.2. The code developed for the implementation of this model is designed to be highly versatile and easy to modify. It can be extended to analyse more complex systems with more treatments and corresponding cell types.

#### A.3.1 Gillespie-like Implementation

The stochastic simulation algorithm (SSA), commonly referred to as the Gillespie algorithm [36], is a Monte Carlo method initially devised in 1977 for modelling the temporal evolution of chemical reactions in a well-mixed system. It has since become an important tool in computational chemistry and systems biology. By simulating specific reactions or events given their rate of occurrence, the Gillespie algorithm mimics the time evolution of a complex system. We implement a version of the Gillespie algorithm for simulating the population dynamics of our system in the context of Extinction Therapy. The idea is that given a set of rates corresponding to birth, death and mutation events, we can track the size of each subpopulation. These rates are specified for all the cell types and can change with time or in response to the environment. The basic steps of our algorithm are as follows:

**Table A.1:** Stopping conditions and corresponding outcomes of a simulation. In the second condition, T is the maximum simulation time defined at the beginning. If we see a significant number of outcomes with persistence (more than 10%), it means that T is not large enough and the simulation is run again with a higher T value. In the third condition, the threshold 0.99K is arbitrary. The outcome remains the same as long as the threshold is close to K. We take the minimum of two quantities for cases where K is much larger than the initial population size, and a threshold of $N_{\max}$ is enough to declare the outcome. Typically, K and $N_{\max}$ are greater than N(0), so persistence in the last condition is not observed. However, the condition is mentioned for the sake of completion.

Stopping condition	Outcome condition	Outcome
N(t)=0	N(t)=0	Extinction
t≥T	N(t)≥N(0)N(t)<N(0)	Progression Persistence
$N(t)\;\geq \;\min(0.99K,\;N_{\max}$)	N(t)≥N(0)N(t)<N(0)	Progression Persistence

1. Initialize the system by specifying an initial population for all cell types $\left(S(0),R_{1}(0),R_{2}(0),R_{1,2}(0)\right)$ and setting the time to zero. Define all possible demographic events and their corresponding rates.
2. Calculate the rate of any one event occurring, which is the sum of all individual rates (denoted by $\omega_{i}(t)$ for each event i). Then, obtain the time interval after which the next event will take place. To do this, generate a sample from an exponential random variable with the rate parameter equal to the total rate $\sum_{i}\omega_{i}(t)$. Alternatively, one can generate a random number $z_{1}$ from the uniform distribution between 0 and 1 and use the following formula to determine the time interval for the next event: $t_{\text{int}}=-ln\left(z_{1}\right)/\sum_{i}\omega_{i}(t)$
3. Calculate event probabilities for the next event by dividing the individual event rates by the total rate: $p_{i}(t)=\omega_{i}(t)/\sum_{i}\omega_{i}(t)$. Use these probabilities to select the next event by generating a random number $z_{2}$ between in 0 and 1. The chosen event k would be the largest j such that $z_{2}-\sum_{i=0}^{j}p_{i}(t)>0$.
4. Implement the chosen event by updating the population of the corresponding cell types. Increment time $t=t+t_{\text{int}}$. For example, if the chosen event is the birth of an $R_{2}$ cell, then $R_{2}\left(t+t_{\text{int}}\right)=R_{2}(t)+1$.
5. Repeat steps 2–4 till a stopping condition is reached.

Note that the effects of carrying capacity and treatment are included while specifying birth and death rates in Section A.3.2. Simulations are stopped under one of three conditions: if the population goes extinct, if it exceeds the maximum simulation time, or if it exceeds some maximum population size. Similarly, the outcome of one run of a simulation can be one of three possibilities: extinction (N(t)=0), progression (N(t)≥N(0)) or persistence (N(t)<N(0)). Note that the stopping conditions do not have an equivalent simulation outcome (see Table A.1).

#### A.3.2 Determining Demographic Event Rates

Following our variant of the Gillespie algorithm, one must define all possible demographic events at the beginning of the simulation and define rates corresponding to those events at each time step. Note that an individual event includes the type of event and the type of cell. For example, a mutation event $S\to R_{1}$ is one individual event and has a rate specified for it. Similarly, the birth of an S cell is a different event than the birth of an $R_{1}$ cell. All simulation parameters used to compute the individual event rates are listed in Table 1.

We derive the birth and death rates for all cell types from the deterministic Logistic model for population growth.

(20)
$$\frac{dM(t)}{dt}=g_{S/R}(t)M(t),$$

where $g_{M}(t)$ is the growth rate of subpopulation $M\in \left\{S,R_{1},R_{2},R_{1,2}\right\}$. The growth rates of sensitive and resistant subpopulations are different,

(21)
$$g_{S/R}(t)=g_{S/R}\left(1-\frac{N(t)}{K}\right)-\delta_{i};\;i=1,2$$

where the total treatment-induced death rate in environment i is $\delta_{i}$ and the presence of this term depends on the sensitivity of different cell types in both environments. For example, the growth rate of $R_{1}$ cells in $E_{1}$ will not include the treatment-induced death term, but in $E_{2}$ will have a $\delta_{2}$ term.

We can write the intrinsic growth rates as the difference between intrinsic birth and death rates like so:

(23)
$$g_{S}(t)\;=(b-d)\left(1-\frac{N(t)}{K}\right)-\delta_{i};\;i=1,2$$

(24)
$$g_{R}(t)=(b-c-d)\left(1-\frac{N(t)}{K}\right)-\delta_{2};$$

where b,d and c are the intrinsic birth rate, death rate and the cost of resistance. Now, we separate the positive and negative parts of the subpopulation growth rates to find the effective birth and death rates to use for our simulations.

(25)
$$b_{S}^{eff}(t)=b-(b-d)\left(\frac{N(t)}{K}\right);\;d_{S}^{eff}(t)\;=d+\delta_{i}$$

(26)
$$b_{R}^{eff}(t)=(b-c)-(b-c-d)\left(\frac{N(t)}{K}\right);\;d_{R}^{eff}(t)=d+\delta_{i}$$

We use Equations 25 and 26 in our simulations. Note that the separation of positive and negative terms in the last step is not unique. We chose this particular way to separate the terms because it follows the condition required for the implementation of carrying capacity, i.e. when the total population is equal to K, we must have $b_{S/R}^{\text{eff}}(t)=d_{S/R}^{\text{eff}}(t)$. Additionally, it is convenient and intuitive to group the death-rate terms together, like we do in the $d_{S/R}^{\text{eff}}(t)$.

For mutation events, we consider the same mutation rate $\mu_{E_{i}}$ for all cell types, which may change with the environment. Once the source population for an event is chosen (say, S cells), the target cell is chosen according to the rates of acquiring each type of mutation. These mutation probabilities from one cell type to the other can be modified.

#### A.3.3 Switching to the second treatment

For this study, we consider two environments $E_{1}$ and $E_{2}$, each corresponding to the two strikes or treatments. Now we ask the question: how does the environment change with time? In other words, what is the condition under which we switch treatments and apply the second strike? This is a very pertinent question because the hypothesis of Extinction therapy relies on the timing of the second strike. However, it is an indirect relation. Extinction therapy aims to exploit the stochasticity of a small population at the time of the second strike. This means that the switch between treatments depends on the population size, which is why we have defined the threshold N(τ) – the population size at which we switch to the second treatment.

It is necessary to understand the behaviour of this variable N(τ) in order to evaluate the efficacy and limitations of Extinction Therapy. Specifically, its relation with the minimum population reached in the absence of the second strike $N_{min}$ is important to explore. As reasoned by Gatenby, Artzy-Randrup, et al. (2020) [18] and supported by our results, the hypothesis is that the optimal N(τ) would be close to $N_{min}$. To test this, we run a set of simulations with the same parameter values but with different random seeds. For a single random seed, we run multiple simulations, each with a different N(τ), all relative to $N_{min}$. This ensures that any differences due to stochasticity are eliminated. To produce Figures 2(C) and 5(C), we run simulations with 100 random seeds for a single set of parameters and then calculate extinction probabilities for various values of N(τ) relative to $N_{min}$. The algorithm is as follows:

1. Select a set of parameter values. These parameters are kept constant throughout.
2. Set random seed for this set of simulations, thus eliminating differences due to stochasticity.
3. Run the first simulation without any second strike. Equivalently, set N(τ) equal to zero for this run. From the results of this run, save the values of $N_{min}$ and its corresponding time point $t\left(N_{min}\right)$.
4. Run the remaining set of simulations with $N(\tau )=mN_{min}$ where the factor m ranges from 1 to a specified value (set to 20 for our simulations). Record the outcomes of all the runs (according to Table A.1. See Figure 2(B) for an illustration.
5. Repeat steps 2–4 for the desired number of random seeds. Each set of simulations with a different random seed is independent and will have different values of $N_{min}$. This means that the absolute N(τ) values will be different for each independent realisation. Therefore, in order to calculate extinction probabilities, we keep the factors m constant for all random seeds.
6. Calculate extinction probabilities using outcomes for each random seed, corresponding to the values of m. We thus obtain extinction probabilities for various values of $N(\tau )/N_{min}$.

### A.4 Metrics to compare treatment outcomes using output from the analytical model

After devising a way to capture the drop in extinction probabilities as N(τ) increases, we wanted to compare these curves for different sets of parameters to determine the optimal conditions for Extinction Therapy. After observing the monotonously decreasing nature of the $N_{q}$ vs q plot, we came up with three metrics to achieve this goal. The first metric, and the one we use in the main text, is the normalised area under the $N_{q}$ vs q curve. A larger area under the curve (AUC) can mean one of two things – first, that the drop in extinction probability occurs at a larger N(τ) or second, that the drop occurs gradually over a larger range of N(τ) values. The first scenario is favourable for ET, and the second one can be beneficial in some cases. If the drop is close to the initial population, then it is easier to implement ET, and if the drop is gradual, then there are chances to obtain high extinction probabilities at high N(τ) values. Clearly, this metric is not very accurate but it is a reasonable choice considering the general trend of $N_{q}$ vs q curves showing a sudden drop in extinction probabilities.

The second metric we propose allows us to find the population size at which this drop occurs. We define the critical second strike threshold as $N_{c}=mean\left(N_{0.1}+N_{0.9}\right)/N(0)$. The higher the $N_{c}$ for a system, the easier it will be to implement extinction therapy. This metric has the advantage of approximating the time of the drop, but it is accurate only if the drop is from a value as high as 0.9 to a value as low as 0.1. Other values for the definition of $N_{c}$ may be considered, but it is hard to find appropriate values that work for a wide range of parameter sets. The third metric is similar to the second one and uses the $N_{0.5}/N(0)$ as a measure of the population size at which this drop occurs.

We compare the three metrics in Figure A.1 and see that they produce similar results.

### A.5 Supplementary Tables and Figures

**Table A2:** population threshold to achieve ≥ 0.8 $P_{E}$

N(0)	δ=1	δ=1.5	δ=2	δ=2.5	δ=3

10^4^	3520.6	3342.8	3314.8	3208.2	3205.6
10^5^	9306.4	6427.9	5647.5	4081.9	4056.4
10^6^	-	15278.6	10474.7	7818.8	6564.3
10^7^	-	-	14747.0	14188.6	11642.3
