# Supplementary material for: Preventing evolutionary rescue in cancer
Source: bioRxiv. 2024 Aug 27:2023.11.22.568336. Originally published 2023 Nov 22. Preprint. [Version 4] doi: 10.1101/2023.11.22.568336 (PMC10690287; doi:10.1101/2023.11.22.568336)
Supplement: Supplement 1 [file NIHPP2023.11.22.568336v4-supplement-1.pdf]

bioRxiv preprint doi: https://doi.org/10.1101/2023.11.22.568336; this version posted August 27, 2024. The copyright holder for this preprint (which was not certified by peer review) is the author/funder, who has granted bioRxiv a license to display the preprint in perpetuity. It is made available under aCC-BY 4.0 International license.

A.9 Supplementary Tables and Figures

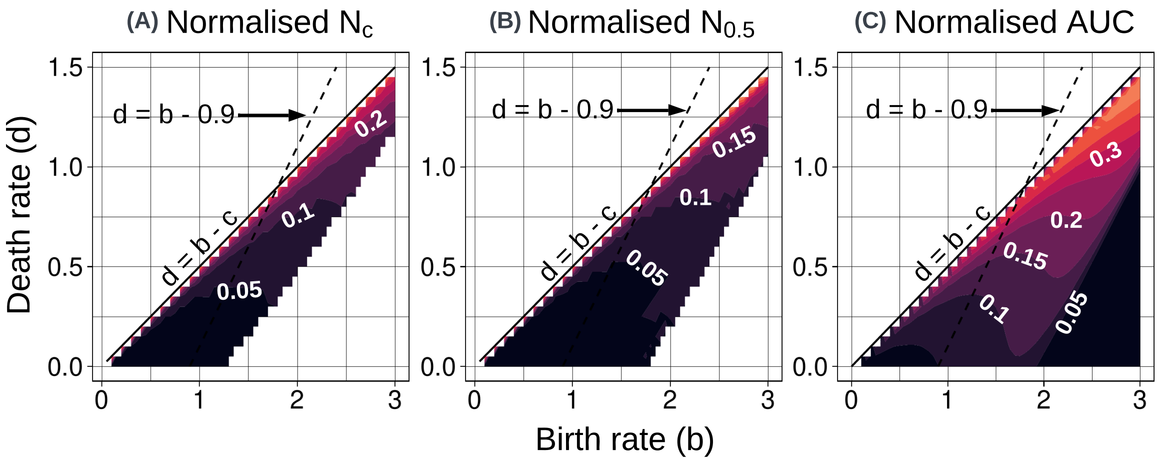

Figure A.1: Comparing treatment outcomes for different parameter values in the $b-d$ space using three different metrics. For each metric, values of birth and death rates are chosen such that the resistant cells have a positive growth rate. The dashed line indicates the set of birth and death rates which result in a constant intrinsic growth rate (equal to the default value of $g_S = 0.9$).**(A)** The metric is defined as the normalised mean of $N_{0.1}$ and $N_{0.9}$. The bottom right region gives invalid values using this metric because $N_{0.9}$ is not defined for those parameters. This is because the optimal extinction probabilities in that region are lower than 0.9. **(B)** The metric is defined as the normalised $N_{0.5}$. Similar to the metric used in plot A, the parameters in the bottom right region give optimal extinction probabilities less than 0.5. **(C)** The normalised area under the curve of the N_q vs q plot for parameters in the $b-d$ space. This metric is favoured because it gives smooth curves on the plot, which also does not contain an invalid region.

bioRxiv preprint doi: https://doi.org/10.1101/2023.11.22.568336; this version posted August 27, 2024. The copyright holder for this preprint (which was not certified by peer review) is the author/funder, who has granted bioRxiv a license to display the preprint in perpetuity. It is made available under aCC-BY 4.0 International license.

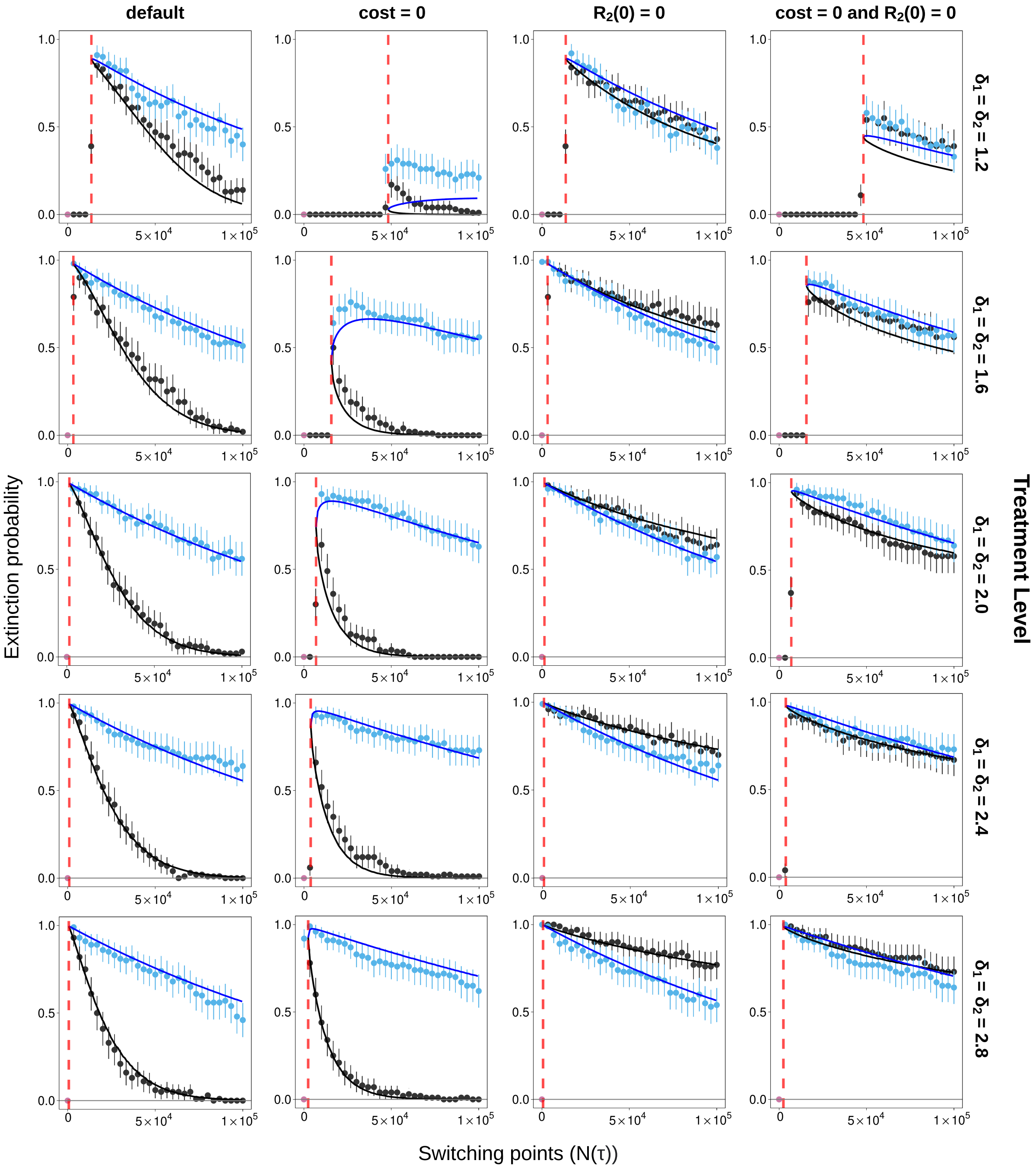

Figure A.2: Simulation results for points of switching before and after $N_{\min}$. For the same random seed, extinction probabilities for different switching points (see Appendix A.6) are plotted. The black points indicate extinction probabilities when the points of switching are before the $N_{\min}$, and blue points indicate $N(\tau)$'s implemented after $N_{\min}$. Pink dots show the extinction probability in the absence of a second treatment. See Figure 2(B) for the legend. All parameters except the cost of resistance, treatment level and initial R_2 population are set to their default values. Error bars show 95% binomial proportion confidence intervals. All extinction probabilities are obtained by considering the outcomes of 100 runs of the simulation with the different random seeds.

bioRxiv preprint doi: https://doi.org/10.1101/2023.11.22.568336; this version posted August 27, 2024. The copyright holder for this preprint (which was not certified by peer review) is the author/funder, who has granted bioRxiv a license to display the preprint in perpetuity. It is made available under aCC-BY 4.0 International license.

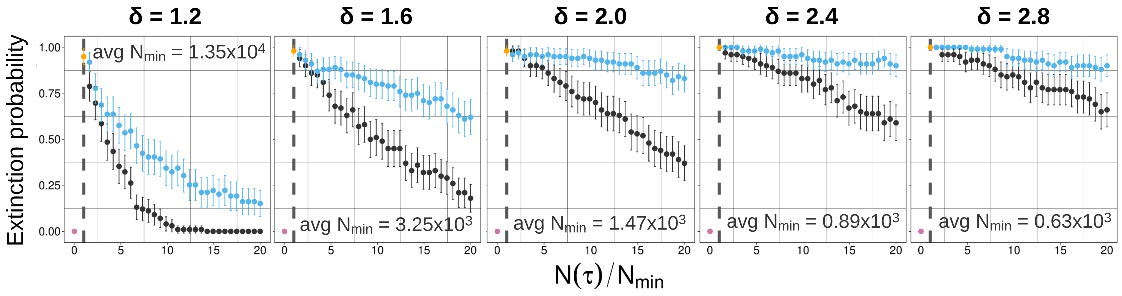

Figure A.3: Extinction probabilities for several switching points relative to the $N_{\min}$ (different for each of 100 independent runs). Black(blue) dots show simulation results for before(after)-nadir switching points.

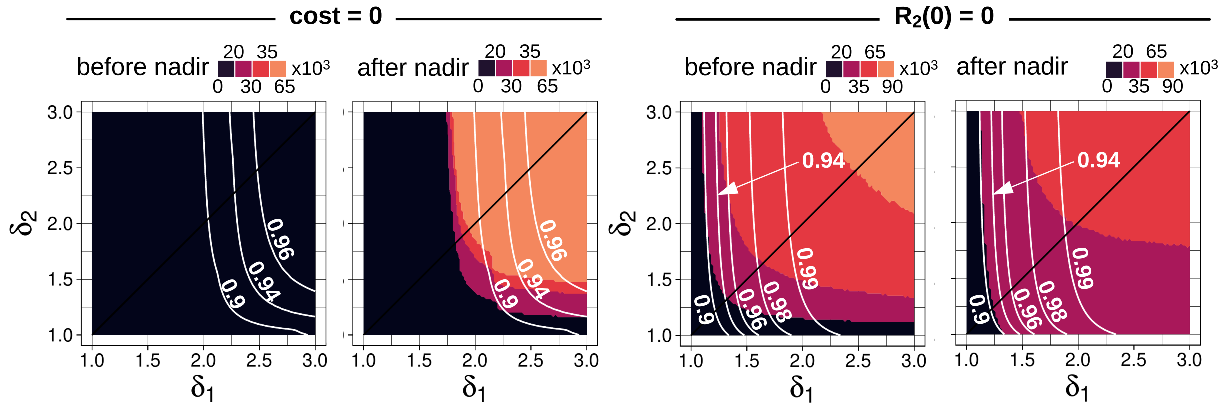

Figure A.4: Heatmaps (obtained from the analytical model) showing the range of $N(\tau)$ values that give a high extinction probability (≥ 0.8) for different combinations of treatment levels δ_1 and δ_2. The case with cost$=0$ is shown on the left, and the case with $R_2(0) = 0$ is on the right. For each case, both before-nadir and after-nadir switching points are considered. White lines indicate optimal extinction probability contours. In the leftmost panel, no high-P_E regions exist.

bioRxiv preprint doi: https://doi.org/10.1101/2023.11.22.568336; this version posted August 27, 2024. The copyright holder for this preprint (which was not certified by peer review) is the author/funder, who has granted bioRxiv a license to display the preprint in perpetuity. It is made available under aCC-BY 4.0 International license.

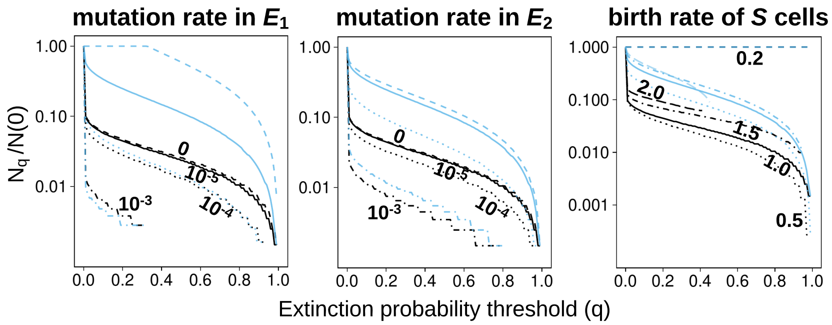

Figure A.5: Normalised N_q vs q plot for different values of total mutation rates in E_1 and E_2, and the intrinsic birth rate. The value of $\mu_{E_1}(\mu_{E_2})$ is kept constant at 10^{-5} when $\mu_{E_2}(\mu_{E_1})$ is varied. Changing the total mutation rate in both environments individually has the same effect (qualitatively). A higher mutation rate results in lower extinction probabilities. This figure is obtained using the analytical model only.

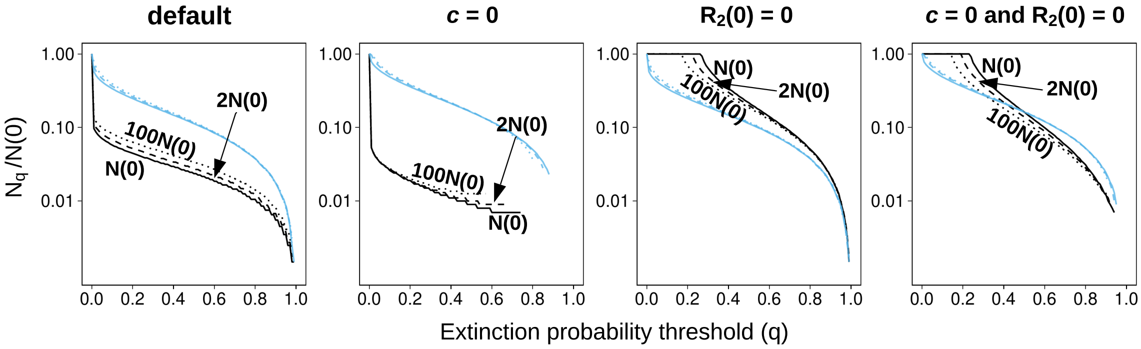

Figure A.6: Normalised N_q vs q plot, obtained using the analytical mode, for different values of the carrying capacity under four conditions – default, no cost of resistance, no initial R_2 population, and the case with $c = 0$ and $R_2(0) = 0$. The last case reveals the effect of changing the carrying capacity in isolation. Black(blue) lines indicate before(after)-nadir switching points. The line type (solid, mixed, dotted) is the same for both before and after nadir curves.

bioRxiv preprint doi: https://doi.org/10.1101/2023.11.22.568336; this version posted August 27, 2024. The copyright holder for this preprint (which was not certified by peer review) is the author/funder, who has granted bioRxiv a license to display the preprint in perpetuity. It is made available under aCC-BY 4.0 International license.

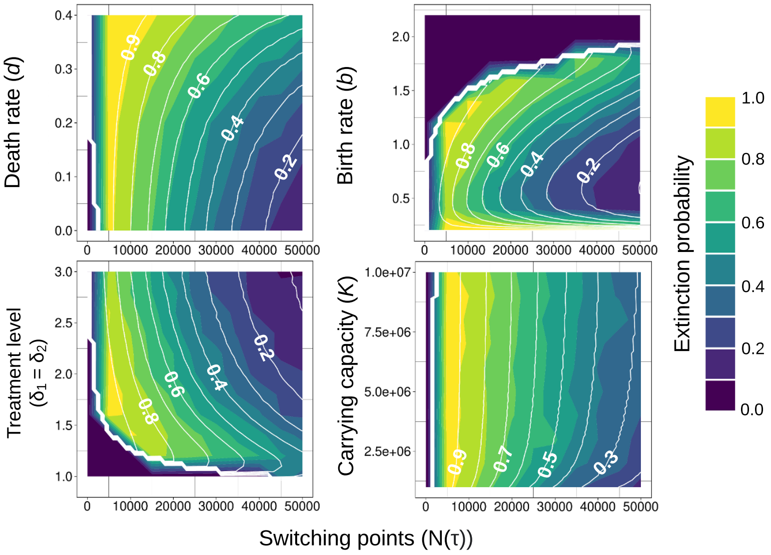

Figure A.7: Extinction probability heatmaps for four system parameters. All other parameters are from the default parameter set (Table 1). In all the heatmaps, the solid white contours (with labels) show analytical results. Stochastic simulation results are denoted by the colour scale. Extinction probabilities from the stochastic model are obtained by using the outcomes of 500 simulations with the same parameter values and initial conditions. In the bottom-left panel, treatment levels for both environments are altered together (δ_1=δ_2). The default treatment level is $\delta = 2.0$ per unit of time. We do not consider treatment levels below 0.9 because that is the intrinsic growth rate of S cells, due to which $\delta < 0.9$ will only give positive growth rates for all cells in the population. The dark regions in the plots (e.g. top-left region in the top-right panel) have low extinction probabilities because $N(\tau) < N_{\min}$ at those points.

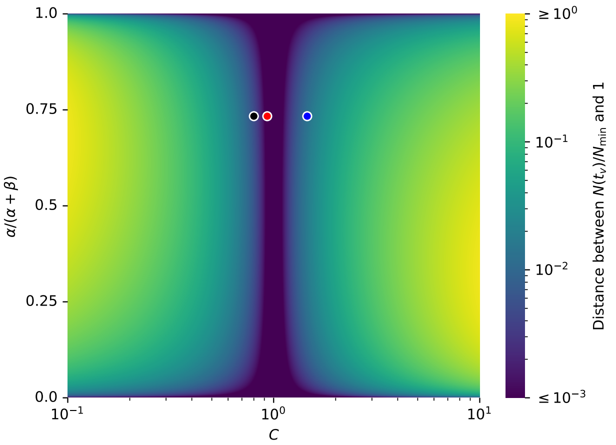

Figure A.8: Measuring how close $\frac{N(t_v)}{N_{\text{min}}}$ (calculated using Lemma A.1) is to 1, where t_v can stand for the time of minimal standing genetic variants (SGV), time of minimal de-novo variants (DN), or time of optimal probability of extinction (opt). α and β were defined in section A.1.2 to be $\delta_1 - \gamma_S$ and γ_1, respectively. C is an axis depending on variables $\delta_2, \gamma_1, c_i, \pi_i$ and its functional form is set to $C = \frac{\gamma_1 \pi_2}{c_2 \pi_{1,2}}$ for $v =$ SGV, $C = \frac{\pi_2/(\delta_2 - \gamma_S)}{\pi_{1,2}/(\delta_2 - \gamma_1)}$ for $v =$ DN, and $C = \frac{\pi_2\left(\frac{1}{\delta_2 - \gamma_S} + \frac{1}{c_2}\right)}{\pi_{1,2}\left(\frac{1}{\delta_2 - \gamma_1} + \frac{1}{\gamma_1}\right)}$ for $v =$ opt (see sections A.1.2.3, A.1.2.2, and A.1.2.4 respectively). The black, blue, and red dots indicate the default parameter choices for $v =$ SGV, DN, opt, respectively.
